# Clinical characteristics of tuberculous meningitis in older patients compared with younger and middle-aged patients: a retrospective analysis

**DOI:** 10.1186/s12879-023-08700-3

**Published:** 2023-10-18

**Authors:** Xiaolin Zhu, Na He, Le Tong, Zhi Han Gu, Hong Li

**Affiliations:** 1 Department of Emergency Medicine, West China Hospital, Sichuan University/West China School of Nursing, Sichuan University, Chengdu, 610041 Sichuan P. R. China; 2 Institute of Disaster Medicine, Sichuan University, Chengdu, 610041 Sichuan P. R. China; 3 Nursing Key Laboratory of Sichuan Province, Chengdu, 610041 Sichuan P. R. China; 4 Emergency Department of West China Hospital of Sichuan University/Emergency Teaching and Research Department of West China Hospital of Sichuan University, Chengdu, 610041 P. R. China

**Keywords:** Tuberculous Meningitis, Clinical characteristics, Older adults

## Abstract

**Background:**

Few studies have analyzed the clinical characteristics and adverse factors affecting prognosis in older patients with tuberculous meningitis (TBM). This study aimed to compare the clinical characteristics of TBM in older patients with those in younger and middle-aged patients.

**Methods:**

This single-center retrospective study extracted data on the clinical features, cerebrospinal fluid changes, laboratory results, imaging features, and outcomes of patients with TBM from patient medical records and compared the findings in older patients (aged 60 years and older) with those of younger and middle-aged patients (aged 18–59 years).

**Results:**

The study included 197 patients with TBM, comprising 21 older patients aged 60–76 years at onset, and 176 younger and middle-aged patients aged 18–59 years at onset. Fever was common in both older (81%) and younger and middle-aged patients (79%). Compared with younger and middle-aged patients, older patients were more likely to have changes in awareness levels (67% vs. 40%), peripheral nerve dysfunction (57% vs. 29%), changes in cognitive function (48% vs. 20%), and focal seizures (33% vs. 6%), and less likely to have headache (71% vs. 93%), neck stiffness on meningeal stimulation (38% vs. 62%), and vomiting (47% vs. 68%). The Medical Research Council staging on admission of older patients was stage II (52%) and stage III (38%), whereas most younger and middle-aged patients had stage I (33%) and stage II (55%) disease. Neurological function evaluated on the 28th day of hospitalization was more likely to show poor prognosis in older patients than in younger and middle-aged patients (76% vs. 25%). Older patients had significantly higher red blood cell counts and blood glucose levels, and significantly lower serum albumin and sodium levels than those in younger and middle-aged patients. The cerebrospinal fluid protein levels, nucleated cell counts, glucose levels, and chloride levels did not differ significantly by age.

**Conclusion:**

In patients with TBM, older patients have more severe clinical manifestations, a higher incidence of hydrocephalus and cerebral infarction, and longer hospital stays than younger and middle-aged patients. Older patients thus require special clinical attention.

## Background

Tuberculous meningitis (TBM) is the most serious form of tuberculosis, resulting in considerable morbidity and mortality in adults and children, with a case fatality rate of up to 50% and an incidence of neurological sequelae after treatment of up to 50% [1]. In recent years, the incidence rate of TBM has increased, especially in middle-aged and older adults, with serious clinical manifestations, poor neurological prognosis, and high disability rate and mortality [2].

In the late stage, TBM can lead to the formation of choroidal plexus and arachnoid adhesions, elevated intracranial pressure, and complications such as cerebral infarction, hydrocephalus, and changes in consciousness and behavior, thus posing a threat to life. A previous clinical study [3] reported that timely and standardized treatment and scientific and careful nursing are key to improving the treatment success rate and reducing the mortality of patients with tuberculosis.

The effect of aging on the clinical manifestations of certain diseases is clear, but information on the clinical characteristics of TBM in older patients is limited, and older patients generally require higher levels of treatment and care. Therefore, this study aimed to compare the clinical characteristics of TBM in older patients with those of younger and middle-aged patients to inform the diagnosis and treatment of TBM in older adults.

## Methods

### Research participants

#### Participants

This retrospective analysis included adult patients admitted to our hospital with TBM from January 1, 2018 to June 1, 2019. The inclusion criteria were age ≥ 18 years and HIV-seronegative results [4]. Pregnant patients, patients with HIV co-infection, and patients with a previous diagnosis of TBM were excluded. The diagnostic criteria were based on the 2010 International Expert Consensus on TBM, which classifies TBM into definite and possible [5], and included the following: (1) chest X-ray (CXR) screening for tuberculosis; (2) fever, headache, vomiting, meningeal symptoms, and other clinical manifestations of meningitis lasting more than 2 weeks; (3) cerebrospinal (CSF) fluid protein level > 1 g/L, CSF to blood glucose ratio < 0.5, and CSF cell count ≥ 20%; (4) focal neurological symptoms and changes in the level of consciousness and cognitive function; and (5) cerebral computed tomography (CT) scan indicating hydrocephalus, cerebral infarction, and basement exudate.

The study was approved by the Ethics Committee of West China Hospital of Sichuan University (approval number 2018 − 598) and was conducted in accordance with the Declaration of Helsinki. The requirement for informed consent to participate in the study was waived due to the retrospective nature of the study.

#### Staging criteria for TBM

The staging of TBM was determined based on the modified Medical Research Council (MRC) criteria [6–8] as follows: Stage I: conscious, only fever, headache, other nonspecific symptoms, and no decreased level of consciousness or neurological signs (Glasgow Coma Scale [GCS] score, 15); Stage II: drowsiness or cranial nerve paralysis (GCS score, 11–14); and Stage III: coma, or paralysis (GCS score ≤ 10).

### Data collection

In this single-center retrospective analysis, all case data were obtained from the electronic medical records of the hospital information system (HIS) of West China Hospital of Sichuan University. We collected clinical data on patients with TBM before and during the most recent hospital admission.

We collected the following data: (1) general demographic data: sex and age; (2) symptoms and signs on admission: fever for > 5 days, cough for > 2 weeks, night sweats, headache, vomiting, nausea, emaciation, convulsions, focal seizures, changes in level of consciousness, and changes in cognitive function; (3) vital signs on admission: body temperature (℃), heart rate (beats/min), respiratory rate (breaths/min), systolic blood pressure (mm Hg), and diastolic blood pressure (mm Hg); (4) physical examination findings: peripheral nerve function defects, cranial nerve paralysis, neck stiffness on meningeal stimulation, and pathological signs; (5) length of hospital stay; (6) laboratory test results (reference range): serum potassium level (3.5–5.5 mmol/L), serum sodium level (135–145 mmol/L), blood glucose level (3.9–6.1 mmol/L), platelet count (100–300 × 10^9^/L), serum albumin level (35–50 g/L), red blood cell count (4.0–5.5 × 10^9^/L), blood leukocyte count (4–10 × 10^9^/L), lymphocyte count (0.8–4 × 10^9^/L), neutrophil absolute value (1.8–6.3 × 10^9^/L), nucleated cell count in the CSF (0–8 × 10^6^/L), CSF protein level (0.15–0.45 g/L), CSF glucose level (2.5–4.4 mmol/L), CSF chloride level (120–130 mmol/L), and CSF/blood glucose ratio ([insert the correct reference value]); (7) imaging findings: head CT or magnetic resonance imaging (MRI) indicating intracranial changes suggestive of cerebral infarction, hydrocephalus, meningeal enhancement, intracranial enhancement lesions, intracranial tuberculoma, and basement membrane exudation; (8) extra-central nervous system tuberculosis: CXR or CT or sputum smear (culture) indicating tuberculosis or extrapulmonary tuberculosis; (9) TBM stage (stage I, II, or III) and the GCS score; and (10) interval from the onset of symptoms to initiation of treatment.

Neurological outcomes were assessed after 28 days through either a ward visit or telephone follow-up. The modified Rankin scale (mRS) score was used as an outcome index to evaluate the patients’ neurological prognosis, with scores of 0–2 indicating a good prognosis, and scores of 3–5 indicating a poor prognosis. The mRS scores were collected by a review of the hospital medical records or telephone follow-up.

### Statistical analysis

All statistical analyses were performed using SPSS version 22.0 (IBM Corp., Armonk, NY, USA). Categorical data were reported as frequencies and percentages. Normally distributed continuous data were reported as the mean ± standard deviation, and non-normally distributed continuous data were expressed as the median and range. In the analysis of outcomes in older patients compared with younger and middle-aged patients, the independent sample *t*-test was performed for comparisons of normally distributed continuous variables, the Wilcoxon rank-sum test was performed for comparisons of non-normally distributed continuous variables, and the chi-square test was performed for comparisons of categorical variables. *P* values < 0.05 were considered statistically significant.

## Results

### Patient characteristics on admission

A total of 197 patients with TBM were included in this study, of whom 21 were in the older age group, with ages ranging from 60 to 76 years and mean age of 66 ± 5.1 years. Of these older patients, 7 (33%) were male and 14 (77%) were female. The remaining 176 patients were in the younger and middle-aged age group, with ages ranging from 18 to 59 years and a mean age of 32.1 ± 12.9 years. Among the younger and middle-aged patients, 102 (58%) were male and 74 (42%) were female.

The mean interval from the onset of symptoms to the diagnosis of TBM was 43.7 ± 76 days in older patients, and 45.8 ± 30.9 days in younger and middle-aged patients. The length of hospital stay was 38.8 ± 23.1 days in older patients, and 23.1 ± 17.5 days in younger and middle-aged patients. The average GCS score was 13.7 ± 1.6 in older patients and 13.6 ± 2.5 in younger and middle-aged patients. Among the older patients, 2 (10%) had stage I, 11 (52%) had stage II, and 8 (38%) had stage III disease. In contrast, among younger and middle-aged patients 59 (33%) had stage I, 98 (55%) had stage II, and 19 (12%) had stage III disease. On the 28th day of hospitalization, 16 (76%) older patients, and 46 (25%) younger and middle-aged patients showed poor neurological status.

Compared with the younger and middle-aged patients, older patients had more advanced disease and were more likely to have a low GCS score (≤ 15 points) at diagnosis, leading to longer hospital stays and worse prognosis at the 28-day assessment due to poor neurological status. The differences in clinical characteristics by age were statistically significant (Table 1).

**Table 1:** Single-factor analysis results of sociodemographic and clinical features in older adults compared with younger and middle-aged adults in patients with TBM

Parameter	Older patients	Younger and middle-aged patients	Statistic	*P*
Sex, *n* (%)				
Male	7 (33)	102 (58)		
Female	14 (77)	74 (42)		
Age, years, mean ± SD	66 ± 5.1	32.1 ± 12.9		
GCS score, divide, mean ± SD	13.7 ± 1.6	13.6 ± 2.5	t = − 0.161	0.872
Delayed treatment time, days, mean ± SD	43.7 ± 76	45.8 ± 30.9	t = 0.121	0.904
Hospital time, days, mean ± SD	38.8 ± 23.1	23.1 ± 17.5	t = − 3.381	0.001
Poor neurological outcome at 28 days, *n* (%)	16 (76)	46 (25)	t = 21.795	0.001
MRC stage, *n* (%)			χ^2^ = 13.73	0.001
Stage I	2 (10)	59 (33)		
Stage II	11 (52)	98 (55)		
Stage III	8 (38)	19 (12)		
Clinical signs on admission, mean ± SD				
Body temperature (℃)	36.8 ± 0.7	37.1 ± 0.9	t = 1.547	0.124
Heart rate (beats/minute)	82 ± 14	89 ± 19	t = 1.614	0.108
Respiratory rate (breaths/minute)	20 ± 2	20 ± 3	t = 0.698	0.486
Systolic blood pressure (mm Hg)	127 ± 23	123 ± 49	t = − 0.408	0.684
Diastolic blood pressure (mm Hg)	78 ± 10	74 ± 15	t = − 0.95	0.343
Symptoms and signs on admission, *n* (%)				
Fever for more than 5 days	17 (81)	139 (79)	χ^2^ = 0.04	0.83
Night sweats	2 (10)	46 (26)	χ^2^ = 2.81	0.09
Cough for longer than 2 weeks	5 (24)	37 (21)	χ^2^ = 0.81	0.76
Headache	15 (71)	165 (93)	χ^2^ = 11.85	0.001
Weight loss	6 (28)	54 (31)	χ^2^ = 0.04	0.84
Vomiting	10 (47)	120 (68)	χ^2^ = 3.53	0.06
Nausea	6 (28)	109 (62)	χ^2^ = 8.59	0.003
Convulsions	5 (24)	29 (16)	χ^2^ = 0.706	0.401
Focal seizures	7 (33)	11 (6)	χ^2^ = 16.57	0.001
Neck stiffness on meningeal stimulation	8 (38)	110 (62)	χ^2^ = 4.65	0.03
Pathology	5 (29)	66 (37)	χ^2^ = 1.52	0.21
Peripheral nerve dysfunction	12 (57)	51 (29)	χ^2^ = 6.84	0.009
Cranial nerve palsy	6 (28)	42 (24)	χ^2^ = 0.22	0.63
Change of consciousness	14 (67)	71 (40)	χ^2^ = 5.3	0.02
Cognitive function change	10 (48)	35 (20)	χ^2^ = 8.187	0.004

### Comparative analysis of vital signs

There were no significant differences in the body temperature, heart rate, respiratory rate, systolic blood pressure, or diastolic blood pressure between the two age groups (Table 1).

### Comparative analysis of clinical characteristics

Compared with the younger and middle-aged patients, older patients had a significantly higher incidence of headache, nausea, changes in the level of consciousness, peripheral nerve function defects, focal seizures, neck stiffness, and cognitive function changes (Table 1). No significant difference was observed in the incidence of fever, night sweats, cough, weight loss, vomiting, convulsions, pathological signs, or cranial nerve paralysis.

### Comparative analysis of laboratory serum test results

The mean serum albumin level was significantly lower in the older age group than in the younger and middle-aged age group. The serum sodium level was also lower in the older age group, but the difference between groups was not statistically significant (*P* = 0.424). No significant difference was noted in the blood potassium level, white blood cell count, lymphocyte count, or absolute neutrophil count between the two age groups. The results of the laboratory tests are summarized in Table 2.

**Table 2:** Single-factor analysis of laboratory, imaging, and sputum smear results in older adults compared with younger and middle-aged adults in patients with TBM

Laboratory parameter	Older group	Younger and middle-aged group	Inspection value	*P*
Blood, mean ± SD				
Serum potassium (mmol/L)	3.62 ± 0.47	3.70 ± 0.46	t = 0.704	0.482
Blood sodium (mmol/L)	131.1 ± 6.2	132.0 ± 8.1	t = 0.802	0.424
Blood glucose (mmol/L)	6.8 ± 2.0	6.0 ± 1.3	t = − 2.31	0.022
Blood platelet (10^9^/L)	197.1 ± 94.8	244.4 ± 99.5	t = 2.067	0.04
Serum albumin (g/L)	34.1 ± 5.5	38.8 ± 4.7	t = 4.232	0.001
Red blood cell count (10^9^/L)	4.07 ± 0.54	4.50 ± 0.58	t = 3.269	0.001
Leukocyte count (10^9^/L)	7.46 ± 3.33	8.01 ± 3.59	t = 0.673	0.502
Lymphocyte count (10^9^/L)	0.99 ± 0.58	1.13 ± 0.74	t = 0.852	0.395
Neutrophil absolute value (10^9^/L)	5.98 ± 3.20	6.24 ± 3.53	t = 0.323	0.747
Cerebrospinal fluid, mean ± SD				
Protein (g/L)	2.52 ± 1.65	3.06 ± 6.21	t = 0.395	0.693
Blood glucose (mmol/L)	1.95 ± 1.00	2.07 ± 1.05	t = 0.509	0.611
Chloride (mmol/L)	112.46 ± 7.47	113.94 ± 8.67	t = 0.752	0.453
Glucose to blood glucose ratio	0.29 ± 0.17	0.36 ± 0.18	t = 1.423	0.156
Nucleated cell count (10^6^/L)	229.85 ± 273.12	220.75 ± 297.33	t = 0.134	0.894
Imaging examination or sputum smear examination, *n* (%)				
Hydrocephalus	15 (71)	53 (30)	χ^2^ = 14.16	0.001
Cerebral infarction	17 (81)	47 (27)	χ^2^ = 25.17	0.001
Meningeal enhancement	9 (43)	58 (33)	χ^2^ = 0.82	0.36
Basement membrane exudation	2 (10)	23 (13)	χ^2^ = 0.213	0.64
Intracranial tuberculoma	2 (10)	4 (2)	χ^2^ = 3.341	0.06
Signs of pulmonary tuberculosis on chest imaging or sputum microscopy or culture	11 (52)	116 (67)	χ^2^ = 1.57	0.45

### Comparative analysis of cerebrospinal fluid examination results

Compared with the younger and middle-aged age group, the older age group had significantly lower mean CSF glucose and chloride levels, significantly lower CSF glucose to blood glucose ratios, and a significantly higher mean nucleated CSF cell count. The older age group also had a higher mean CSF protein level; however, the CSF protein level did not differ significantly by age group (Table 2).

### Comparative analysis of imaging examinations or sputum smears

Compared with the younger and middle-aged patients, older patients had a significantly higher incidence of hydrocephalus and cerebral infarction, whereas the incidence of basement membrane exudation, meningeal enhancement, and intracranial tuberculosis did not differ significantly by age. The imaging features are compared in Table 2.

## Discussion

TBM is a non-suppurative inflammatory disease and is the most serious type of tuberculosis. We compared the clinical characteristics of older patients with TBM with those of younger, and middle-aged patients with TBM. Compared with younger and middle-aged patients, the 21 older patients with TBM were more likely to have acute onset, complex disease, rapid progression, multiple complications, and had a higher mortality rate. The most common symptoms of TBM are fever (81%) and headache (71%), and the most common disease manifestations are cerebral infarction (81%), hydrocephalus (71%), neck stiffness(38%), focal seizures (33%), and seizures (24%) [9, 10].

Owing to the high risk and concealed nature of onset, accidents during treatment can pose a serious threat to the life and safety of patients [11]. Therefore, patients should be closely monitored. Clinicians should not only consider the common complications caused by pulmonary tuberculosis (52%), but also remain vigilant for the possibility of intracranial tuberculosis infection. Rapid diagnostic workup including laboratory, CSF, and imaging examinations is necessary for early diagnosis of the disease. Specialized timely treatment and meticulous care should also be provided to control the clinical manifestations, relieve symptoms, and improve patients’ quality of life.

We found that older patients exhibited distinct characteristics compared with younger and middle-aged patients, including a higher clinical stage, poorer prognosis, and longer hospital stays [12]. The proportion of patients with stages II and III TBM in the older group was significantly higher than that in the younger and middle-aged group, similar to the findings of previous studies [13, 14]. Another study reported that the higher the stage, the higher the mortality rate [15].

The average hospitalization time was 38 days in older patients, and 23 days in younger and middle-aged patients, and patients with hospital stays longer than 28 days had poor neurological outcomes, regardless of their age. The findings that older patients had longer hospital stays and worse neurological outcomes is consistent with the results of previous studies [16, 17]. This study confirmed that in patients with TBM, older patients have more severe disease, with higher disability and mortality rates than younger and middle-aged patients.

The incidence of cognitive dysfunction and peripheral nerve function defects is significantly higher in older patients with TBM than in younger and middle-aged patients [18]. In recent years, with the rapid aging of the population in China, the incidence rate of TBM in older adults has increased annually. Owing to the many symptoms of TBM and the varying levels of education and occupation in the population, there is a lack of relevant knowledge about the disease. Consequently, patients with TBM are susceptible to experiencing negative emotions, and the side effects of medication can negatively impact patient adherence to treatment, thereby causing disease progression and decreasing the quality of life, ultimately leading to poor response to treatment. Therefore, meticulous and careful nursing interventions must be adopted during the treatment of older patients to improve treatment effectiveness and prognosis [19]. Routine care, which primarily focuses on preventing intracranial complications and cross-infection and ensuring adherence to treatment, is often not implemented appropriately and fails to fully address the psychological needs of patients, resulting in a high incidence of early complications. In older patients with TBM, it is necessary to provide detailed nursing care, which encompasses a humanized nursing model. Cognitive dysfunction and peripheral nerve function defects impair body functions and reduce self-care ability. A safe diagnostic and treatment environment should be provided, with frequent monitoring, close observation, and timely problem detection and management, to help patients develop confidence in overcoming the disease and meet their psychological and physiological needs. This approach aims to reduce the incidence of complications, achieve an optimal prognosis, and return the patient to the family and society as soon as possible [20].

Compared with the younger and middle-aged group, the older group was more likely to experience changes in the level of consciousness. Due to changes in the level of consciousness, they may experience loss of appetite or not be able to eat normally. Coupled with symptoms of vomiting and nausea, this can lead to low blood sodium and protein levels, electrolyte disorders, and malnutrition. In the older patients with TBM, the mean blood sodium and serum albumin levels were lower than the normal levels. Hyponatremia significantly affects the prognosis of patients with TBM, especially when it is moderate to severe, as it is associated with multiple complications and poor prognosis. In severe cases, it can cause life-threatening brain edema and herniation [21]. In older patients with TBM complicated by hyponatremia, it is crucial to correct the hyponatremia promptly to improve prognosis and reduce mortality. Methods such as peripheral and central venous catheter fluid replacement therapy and enteral nutrition should be used, with careful monitoring of blood biochemistry. Based on the test results, treatment and nursing plans should be adjusted to reduce the occurrence of complications [22]. A previous study found that the prevalence of hyponatremia in patients with TBM was 52%, and the mortality rate was 29% [23], therefore hyponatremia requires clinical attention.

Patients with TBM have a high prevalence of comorbidities and a high incidence of complications, leading to poor prognosis. Therefore, in cases of unclear diagnosis, CSF cytology can serve as a useful tool for timely diagnosis of patients with signs of meningitis, which can help improve the effectiveness of treatment. In patients with suspected TBM, microbial evidence should always be sought, and treatment should begin as soon as possible. Cerebrospinal fluid cytology examination is important for the diagnosis, monitoring, and evaluation of the prognosis of TBM [24].

Intracranial lesions in older patients are prone to cerebral infarction (81%), hydrocephalus (71%), meningeal enhancement (43%), intracranial tuberculosis (10%), and basement membrane exudation (10%) [25]. Headache, nausea, and vomiting are manifestations of elevated intracranial pressure, with elevated intracranial pressure as the most common symptom [25]. The main cause is hydrocephalus [26–28], which can cause brain edema and exacerbate intracranial hypertension in patients. Prolonged elevation of intracranial pressure can lead to ventricular displacement and cerebral herniation, leading to coma and death. In patients with TBM, the combination of hydrocephalus and elevated intracranial pressure generally indicates that the patient’s condition is critical [29, 30]. Early identification of patients with intracranial hypertension and accurate determination of lesion location, lesion type, and extent using head imaging CT or MRI are important for determining the disease severity [31, 32].

This study has some limitations. As it was a retrospective, single-center study with a small number of older patients, further multicenter studies with larger sample sizes are needed to confirm these findings. Nevertheless, this study provides valuable insights into the treatment of older patients with TBM.

## Conclusions

In patients with TBM, older patients require longer length of hospital stay and are at greater risk of developing complications such as extracranial tuberculosis, pulmonary infection, cerebrovascular disease, and electrolyte disorders than younger and middle-aged patients. TBM manifests as subacute or chronic disease with atypical and complex clinical manifestations. Early laboratory CSF examination and MRI examination are helpful for the diagnosis of TBM in older adults. The main clinical manifestations include fever, headache, nausea, changes in the level of consciousness, peripheral nerve function defects, focal seizures, neck stiffness, and cognitive function changes. Atypical symptoms include night sweating, coughing, weight loss, vomiting, convulsions, pathological signs, and cranial nerve paralysis. Most older adults have MRC stage II or III disease at the time of diagnosis, and the arachnoid membrane, brain parenchyma, and cerebral blood vessels are often affected. The condition is severe, leading to extended hospital stays and poor neurological outcomes. Complications frequently occur, resulting in high disability and mortality rates.

Brain imaging examination is useful for detecting hydrocephalus, cerebral infarction, meningeal enhancement, and tuberculoma. Thus, clinicians should pay attention to the atypical clinical manifestations of TBM, closely observe the changes in cerebrospinal fluid and imaging manifestations, and comprehensively analyze the changes in the patient’s condition. When TBM is clinically suspected, it is necessary to take a detailed medical history, evaluate MRC staging in a standardized manner, administer MRI contrast-enhanced scanning, and initiate standard antituberculous treatment early to avoid diagnosis and treatment in stages II and III, minimize patient pain, and reduce the social and economic burden. Early diagnosis, timely treatment, and meticulous care are the key to improving the prognosis of the disease.

## Data Availability

The datasets used and/or analyzed during the current study are available from the corresponding author on reasonable request.

## List of abbreviations

CSF: Cerebrospinal fluid
CT: Computed tomography
CXR: Chest X-ray
GCS: Glasgow Coma Scale
HIS: Hospital information system
MRC: Medical Research Council
MRI: Magnetic resonance imaging
mRS: Modified Rankin scale
TBM: Tuberculous meningitis
